# The Disruption of the β-Catenin/TCF-1/STAT3 Signaling Axis by 4-Acetylantroquinonol B Inhibits the Tumorigenesis and Cancer Stem-Cell-Like Properties of Glioblastoma Cells, In Vitro and In Vivo

**DOI:** 10.3390/cancers10120491

**Published:** 2018-12-05

**Authors:** Heng-Wei Liu, Yu-Kai Su, Oluwaseun Adebayo Bamodu, Dueng-Yuan Hueng, Wei-Hwa Lee, Chun-Chih Huang, Li Deng, Michael Hsiao, Ming-Hsien Chien, Chi-Tai Yeh, Chien-Min Lin

**Affiliations:** 1 Graduate Institute of Clinical Medicine, College of Medicine, Taipei Medical University, Taipei City 11031, Taiwan; henryway0404@hotmail.com (H.-W.L.); yukai.su@gmail.com (Y.-K.S.); mhchien1976@gmail.com (M.-H.C.); 2 Department of Neurology, School of Medicine, College of Medicine, Taipei Medical University, Taipei City 11031, Taiwan; 3 Division of Neurosurgery, Department of Surgery, Taipei Medical University-Shuang Ho Hospital, New Taipei City 23561, Taiwan; 4 Taipei Neuroscience Institute, Taipei Medical University, Taipei 11031, Taiwan; dr_bamodu@yahoo.com; 5 Department of Medical Research & Education, Taipei Medical University-Shuang Ho Hospital, New Taipei City 23561, Taiwan; 6 Department of Neurological Surgery, Tri-Service General Hospital, National Defense Medical Center, Taipei 11490, Taiwan, ROC; hondy2195@yahoo.com.tw; 7 Department of Pathology, Taipei Medical University-Shuang Ho Hospital, Taipei 23561, Taiwan; whlpath97616@s.tmu.edu.tw; 8 Department of Applied Chemistry, Chaoyang University of Technology, Taichung 41147, Taiwan; john@newbellus.com.tw; 9 Beijing Bioprocess Key Laboratory, College of Life Science and Technology, Beijing University of Chemical Technology, Beijing 100029, China; dengli@mail.buct.edu.cn; 10 Amoy-BUCT Industrial Bio-technovation Institute, Amoy 361022, China; 11 Genomics Research Center, Academia Sinica, Taipei 11529, Taiwan; mhsiao@gate.sinica.edu.tw

**Keywords:** glioblastoma, β-catenin, cancer stem cell, 4-AAQB, chemoresistance, prognosis, survival

## Abstract

Background: Glioblastoma (GBM), a malignant form of glioma, is characterized by resistance to therapy and poor prognosis. Accumulating evidence shows that the initiation, propagation, and recurrence of GBM is attributable to the presence of GBM stem cells (GBM-CSCs). Experimental approach: Herein, we investigated the effect of 4-Acetylantroquinonol B (4-AAQB), a bioactive isolate of *Antrodia cinnamomea*, on GBM cell viability, oncogenic, and CSCs-like activities. Results: We observed that aberrant expression of catenin is characteristic of GBM, compared to other glioma types (*p* = 0.0001, log-rank test = 475.2), and correlates with poor prognosis of GBM patients. Lower grade glioma and glioblastoma patients (*n* = 1152) with low catenin expression had 25% and 21.5% better overall survival than those with high catenin expression at the 5 and 10-year time-points, respectively (*p* = 3.57e-11, log-rank test = 43.8). Immunohistochemistry demonstrated that compared with adjacent non-tumor brain tissue, primary and recurrent GBM exhibited enhanced catenin expression (~10-fold, *p* < 0.001). Western blot analysis showed that 4-AAQB significantly downregulated β-catenin and dysregulated the catenin/LEF1/Stat3 signaling axis in U87MG and DBTRG-05MG cells, dose-dependently. 4-AAQB–induced downregulation of catenin positively correlated with reduced Sox2 and Oct4 nuclear expression in the cells. Furthermore, 4-AAQB markedly reduced the viability of U87MG and DBTRG-05MG cells with 48 h IC_50_ of 9.2 M and 12.5 M, respectively, effectively inhibited the nuclear catenin, limited the migration and invasion of GBM cells, with concurrent downregulation of catenin, vimentin, and slug; similarly, colony and tumorsphere formation was significantly attenuated with reduced expression of c-Myc and KLF4 proteins. Conclusions: Summarily, we show for the first time that 4-AAQB suppresses the tumor-promoting catenin/LEF1/Stat3 signaling, and inhibited CSCs-induced oncogenic activities in GBM in vitro, with in vivo validation; thus projecting 4-AAQB as a potent therapeutic agent for anti-GBM target therapy.

## 1. Introduction

Glioblastoma (GBM), one of the most lethal malignancies in adults, with an average incidence of 3.2/100 000, median survival of about 15 months, and median age at diagnosis of 64 years, is the most common and highly aggressive brain tumor [1]. Histopathologically-defined by necrosis and endothelial proliferation, GBM are high-grade (World Health Organization (WHO) grade IV) gliomas which are characteristically resistant to anticancer therapy, resulting in death within 12 months of diagnosis or initiation of therapy [2]. Glioblastoma is characterized by sustained self-renew potential, enhanced tumorigenicity and invasiveness, high likelihood to relapse, and increased resistance to chemotherapy; all features associated with the presence and activities of a small tumor subpopulation called cancer stem cells (CSCs) or tumor-initiating cells (TICs) and referred to herein as GBM stem cells (GBM-SCs). Current anti-GBM therapeutic strategy consisting of concurrent chemoradiotherapy (CCRT) with the DNA alkylating agent, temozolomide (TMZ), is plagued by eventual disease relapse and only prolongs the median survival to 14.6 months [3], necessitating the discovery and/or development of new therapeutic strategy with high anti-GBM efficacy.

In their published review, Vogelstein et al. [4] analyzed several alterations in the human cancer genome and identified tumor driver or promoter genes, as well as outlined twelve associated signaling pathways including Notch, Hedgehog (Hh), and Wnt/β-catenin, known to regulate the determination of cell fate, survival, proliferation, and genome stability. Though infrequently mutated in GBM, these pathways are often aberrantly activated and enhance the CSC-like phenotypes of GBM cells [5]. While our understanding of the molecular mechanism underlying the pathogenesis of GBM is incomplete and continues to evolve, as suggested above, there is increasing evidence that GBM-SCs play an important role in gliomagenesis, tumor maintenance and subsequent disease progression [5], and that Wnt/β-catenin signaling is implicated in the modulation of GBM-SCs [6]. Consistent with this, in a recent study, FoxM1 was shown to enhance the nuclear localization of β-catenin, regulate the expression of target genes of Wnt and gliomagenesis; similarly, close correlation was demonstrated between Wnt/β-catenin signaling, GBM progression, and patient prognosis [7], as well as cancer stemness, self-renewal, and resistance to therapy [8,9], thus, reinforcing the notion that the targeting of Wnt/β-catenin-mediated oncogenicity, self-renewal, and therapy-resistance may represent a critically efficacious anti-GBM treatment strategy.

The principal factor in the canonical Wnt signaling pathway is β-catenin. Nuclear β-catenin facilitates activation of T-cell factor/lymphoid enhancing factor (TCF/LEF) transcription factors, consequently modulating tumor formation, cell-cycle progression, cell survival, and stem-cell like activities [5], thus, making β-catenin a potential therapeutic target in anti-GBM therapy.

In the present study, understanding that the aberrant activation of the Wnt signaling is implicated in gliomagenesis, mediates the maintenance of GBM-SCs and enhances GBM invasive potential [6], as well as building on the demonstrated anticancer therapeutic efficacy of the relatively novel phytochemical 4-acetylantroquinonol B (4-AAQB), a derivative of mono-acetylated antroquinonol, which is a bioactive extract of *Antrodia camphorata* [10,11], we hypothesized and investigated if and how 4-AAQB through the downregulation of β-catenin expression and/or activity, can inhibit the activation of β-catenin-modulated genes in GBM cell lines, U87MG and DBTRG-05MG. The plausibility of this hypothesis is rooted in our previous demonstration of the ability of 4-AAQB to suppress autophagic flux and improves cisplatin sensitivity in highly aggressive epithelial cancer through the PI3K/Akt/mTOR/p70S6K signaling pathway [11], coupled with our understanding that the inhibition of autophagy by mTORC1, a complex of mTOR, rescues disheveled (Dvl), which is key component of Wnt signaling, and thus lead to the activation of Wnt/β-catenin pathway [12]. In addition, we examined the role of 4-AAQB in modulating the responsiveness of GBM cells to anticancer therapy. Thus, we present a novel anti-GBM therapeutic approach by inhibiting the oncogenic Wnt signaling through direct β-catenin targeting by the 4-AAQB.

## 2. Materials and Methods

### 2.1. Drugs and Chemicals

4-acetylantroquinonol B (4-AAQB; >99% HPLC purity) was purchased from New Bellus Enterprises Co., Ltd (Tainan, Taiwan). Stock solution of 1 mM dissolved in dimethyl sulfoxide (DMSO; Sigma–Aldrich Co., St. Louis, MO, USA) was stored at −20 °C then further diluted in sterile culture medium immediately prior to use. Gibco^®^ RPMI-1640, fetal bovine serum (FBS), Trypsin/EDTA, DMSO, phosphate buffered saline (PBS), sulforhodamine B (SRB) medium, Acetic acid, and TRIS base were also purchased from Sigma-Aldrich Co. (St. Louis, MO, USA).

### 2.2. Cell Lines and Culture

The human GBM cell lines U87MG and DBTRG-05MG were obtained from American Type Culture Collection (ATCC, Manassas, VA, USA). Cells were cultured in RPMI-1640, supplemented with 10% FBS and 1% penicillin/streptomycin (Invitrogen, Life Technologies, Carlsbad, CA, USA) and incubated in 5% humidified CO_2_ incubator at 37 °C. The cells were passaged at 95% confluence or culture medium changed every 72 h. For drug cytotoxicity assays, the cells were treated with different concentrations of 4-AAQB for different duration.

### 2.3. Sulforhodamine B (SRB) Cell Viability Assay

U87MG or DBTRG-05MG cells were seeded in supplemented media at a density of 3.5 × 10^3^ cells/well in triplicates in 96-well plates. After 24 h incubation, cells were treated with different concentrations of 4-AAQB. After 24 h or 48 h of treatment, the treated cells were washed in PBS, fixed with 10% trichloroacetic acid (TCA) for 1 h, washed with distilled water, and the viable cells incubated in 0.4% SRB (*w*/*v*) in 1% acetic acid at room temperature for 1 h. The unbound dye was removed by 1% acetic acid washing thrice and the plates air-dried. Attached dye was dissolved in 10mM trizma base, and absorbance was read in a microplate reader at a wavelength of 570 nm.

### 2.4. Western Blot Analysis

Ten-μg protein samples were run in 10% SDS-PAGE gel and transferred onto a polyvinylidene fluoride (PVDF) membrane using the Bio-Rad Mini-Protean system (Bio-Rad Laboratories, Inc, Hercules, CA, USA). Non-specific binding was blocked by incubating the membranes in 5% skimmed milk in tris-buffered saline with Tween 20 (TBST) for 1 h and then overnight at 4 °C with the antibodies against total β-catenin (1:1000, Cell Signaling Technology, Danvers, MA, USA), free β-catenin (1:1000, Cell Signaling Technology), p-GSK-3β (1:1000, Cell Signaling Technology), GSK-3β (1: 1000, Cell Signaling Technology), TCF1/TCF7 (1:1000, Cell Signaling Technology), LEF1 (1:1000, Cell Signaling Technology), p-Stat3 (1:1000, Santa Cruz, California, USA), Stat3 (1:1000, Santa Cruz), KLF4 (1:1000, Santa Cruz), c-Myc (1:1000, Santa Cruz), vimentin (1:1000, Cell Signaling Technology), Slug (1:1000, Cell Signaling Technology), and β-actin (1:500, Santa Cruz). After overnight probing with primary antibody, the membranes were incubated with horseradish peroxidise (HRP)-linked secondary antibodies for 1 h, then washed with PBS thrice. The protein band signals were detected and developed using an enhanced chemiluminescence (ECL) detection system (Thermo Fisher Scientific Inc, Waltham, MA, USA). Protein bands were quantified using ImageJ software.

### 2.5. β-catenin siRNA Infection

For β-catenin loss of function assays, U87MG or DBTRG-05MG cells were infected with stealth siRNAs targeting CTNNB1 (HSS102461; Thermo Fisher Scientific Inc., Lai Fu Life Science and Technology Co., Ltd. Taipei, Taiwan) according to the manufacturers’ protocol. Briefly, the cells at 70–75% confluence per well in 6-well plates were infected with 30 nmol/L siRNA or control/mock siRNA and 3 μL RNAiMAX reagent (Thermo Fisher Scientific Inc., Lai Fu Life Science and Technology Co., Ltd. Taipei, Taiwan). The infection efficiency was confirmed using microscopy and Western blot assays before being used in this study.

### 2.6. Immunohistochemical Staining and Quantification

Paraffin-embedded primary or recurrent GBM tissue sections were used for examination of β-catenin expression, as well as hematoxylin and eosin (H&E) staining. For Immunohistochemistry (IHC) assay, sections were incubated with primary anti- β-catenin (Cell Signaling Technology 1:200 dilutions) at 4 °C overnight, followed by a biotin-labeled secondary antibody (1:100 dilutions) at room temperature for 1 h. Sections were incubated in ABC-peroxidase and diaminobenzidine (DAB), counterstained with hematoxylin and visualized using light microscopy. For Immunofluorescence staining and analysis, the cells were plated in 6-well chamber slides (Nunc, Thermo Fisher Scientific, Taipei, Taiwan) at 4 °C overnight, fixed in 2% paraformaldehyde at room temperature for 10 min, then permeabilized with 0.1% Triton X-100 in 0.01 M phosphate-buffered saline (PBS), pH 7.4 containing 0.2% bovine serum albumin (BSA). Thereafter, they were air-dried and rehydrated in PBS. Followed by incubating the cells with antibody against β-catenin (D10A8; XP Rabbit mAb #8480; Cell Signaling Technology), Sox2 (D6D9; XP Rabbit mAb #3579; Cell Signaling Technology), Oct4 (C30A3; Rabbit mAb #2840; Cell Signaling Technology), or F-actin (13E5; Rabbit mAb #4970; Cell Signaling Technology) diluted 1:500, in PBS containing 3% normal goat serum at room temperature for 2 h. For negative controls we omitted the primary antibody. After PBS washing twice for 10 min each, anti-rabbit IgG fluorescein isothiocyanate-conjugated secondary antibody (Jackson Immunoresearch Lab. Inc., West Grove, PA, USA) diluted 1:500 in PBS was added, and the cells incubated at room temperature for 1 h. This was followed by PBS washing and cell mounting using the Vectashield mounting medium and 4′, 6′-diamidino-2-phenylindole (DAPI) to counter stain DNA for nucleus visualization. Cells were observed under a Zeiss Axiophot (Carl Zeiss, Strasse, Oberkochen Germany) fluorescence microscope. Immunohistochemistry in the evaluation was performed by 2 independent pathologists who were blinded to the study. Immunohistochemistry in the evaluation was performed by 2 independent pathologists who were blinded to the study. The staining results were classified into 3 categories based on β-catenin subcellular localization, namely nuclear, cytoplasmic or membranous. All slides were assessed under microscope and initial score representing the estimated proportion (0: ≤ 5%, 1: 5%–25%, 2: 25%–75%, and 3: ≥ 75%) of positively stained (P) cancer cells, while the intensity (I) scores 1, 2, or 3 were assigned to weak, moderate, and strong stainings, respectively. Slides with indeterminate evaluation were re-assessed until consensus was reached. The Quick score (Q) was calculated by multiplying the percentage of positive cells (P) by the intensity (I) based on the formula below:

Q =P×I; maximum=300

### 2.7. Tumorsphere Formation Assay

For tumorsphere generation, U87MG and DBTRG-05MG cells were cultured in HEScGRO^TM^ serum-free medium for human embryonic stem cell culture (Chemicon, SCM020; Merck KGaA, Darmstadt, Germany), supplemented with 10 ng/mL hbFGF (Invitrogen, Carlsbad, CA), 20 ng/mL hEGF (Millipore, Bedford, MA), B27 supplement (Invitrogen, Carlsbad, CA), heparin (#07980; STEMCELL Technologies Inc., Interlab Co., Ltd, Taipei, Taiwan), and NeuroCult^TM^ NS-A proliferation supplement (Human; #05753; STEMCELL Technologies Inc. Interlab Co., Ltd, Taipei, Taiwan). Cells were seeded at 1000 cells/mL/well in 6-well ultra-low adhesion plates (Corning Inc., Corning, NY, USA) and cultured for 7–10 days. The non-adherent tumorspheres (≥ 90 m in diameter) were viewed, counted, and photographed under inverted phase contrast microscope.

### 2.8. Colony Formation Assay

Two × 10^4^ U87MG or DBTRG-05MG cells were seeded per well in triplicate in 6-well culture plates with or without indicated concentration of 4-AAQB and incubated at 37 °C in a 5% CO_2_ atmosphere incubator for 12–14 days. Generated colonies with diameter ≥ 100 μm and consisting of ≥50 cells, were washed two times with PBS, fixed with methanol for 15 min, and stained with 0.005% crystal violet for 15 min at room temperature for visualization and counting of colonies. The number and size of colonies formed were estimated with the ChemiDoc-XRS imager (QuantityOne software package; Bio-Rad, Hercules, CA, USA).

### 2.9. Invasion Assay 

For the invasion assay, 24-well plate Transwell systems were used; 3 × 10^4^ U87MG or DBTRG-05MG cells were seeded into the upper chamber of the insert (BD Bioscience, 8 μm pore size) containing media without serum, while media containing 10% FBS in the lower chamber was used as chemo-attractant. Media was discarded after incubating for 24 h, and the GBM cells on the filter membrane were fixed for 1 h with 3.7% formaldehyde, before staining with crystal violet dye, and cells lying on the upper surface of the insert were cleared off using a cotton swab. Visualization of the migrated cells and evaluation of their migratory capacity based on the total number of cells on the lower surface of the filter membrane was performed under microscope.

### 2.10. Wound-healing Migration Assay 

The GBM cell lines, U87MG or DBTRG-05MG, were seeded in 6-well plates and incubated in 5% CO_2_ atmosphere incubator for 48 h until they were 100% confluent. Scratch wounds of similar sizes were then made along the median axis of the adherent cells using sterile 200 µL micropipette tips. After PBS washing to remove detached cells, adherent cells were incubated in new culture medium in 5% CO_2_ humidified incubator at 37 °C to allow wound to close. Healing/closure of wound-gap was monitored and photographed at the indicated time points.

### 2.11. Animal Studies

Six–8-weeks old female Non-Obese Diabetic (NOD)/Severe-Combined Immunodeficient Mice (SCID) mice (*n* = 15) obtained from BioLASCO Taiwan Co., Ltd (Taipei, Taiwan) were bred under standard experimental animals specific-pathogen-free conditions. The mice (5/treatment group) were subcutaneously inoculated on the right flank with 0.5 × 10^6^ U87MG cells in 0.5 ml PBS. Treatment was started on day 7–10 when tumors reached an average size of ≥150 mm^3^. Treatment group 1 consisted of thrice weekly intraperitoneal (i.p.) injections of 5 mg/kg 4-AAQB in 0.5 ml PBS for 4 weeks; Treatment group 2 consisted of thrice weekly oral (p.o.) gavage of 5 mg/kg 4-AAQB in 0.5 ml PBS for 4 weeks; while the control group were injected with PBS. Tumor growth was measured two times per week using calipers, and tumor volume (v) calculated using the formula: v = (width)^2^ × length/2. Animals were followed for another 3 weeks after the final 4-AAQB treatment (i.e., 7 weeks after tumor inoculation), then the treated and control with extremely large tumors were humanely sacrificed. Mice with small tumors were allowed for an additional 8–12 weeks. Assessment of tumor growth for each of the studies was carried out and statistical analyses was determined by Student’s *t*-test using Sigma plot v13 (Stystat Software, CA, U.S.A.). *p* < 0.05 was considered statistically significant. All the experimental animal procedures were approved by and performed in accordance to the Institutional Animal Care and Use Committee/Panel (IACUC/P) protocol approval LAC-2015-0386.

### 2.12. Statistical Analysis

Each experiment was performed at least 4 times in triplicates. Shown data represent means ± SD. Comparison between two groups was estimated using the 2-sided Student’s *t*-test, while the one-way analysis of variance (ANOVA) was used for comparison between 3 or more groups. *p*-value < 0.05 was considered statistically significant.

## 3. Results

### 3.1. Aberrant Expression of β-Catenin Is Characteristic of GBM and Correlates with Poor Prognosis

**Figure 1:**
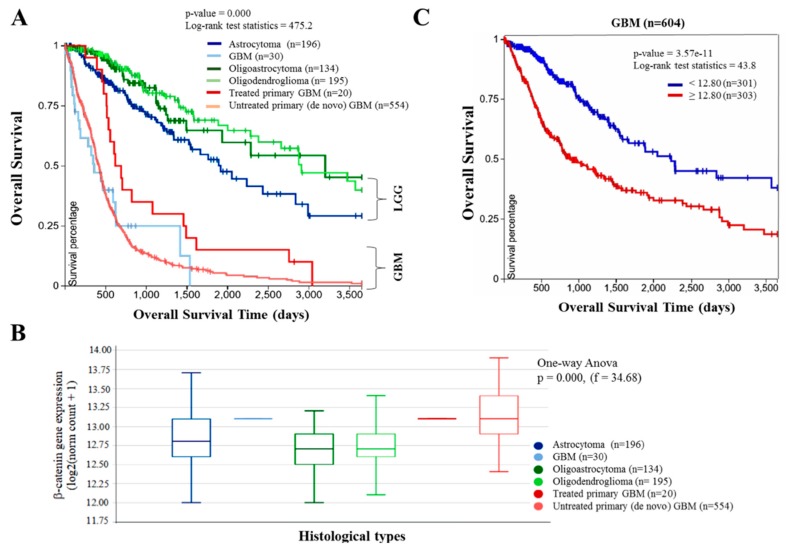
Aberrant expression of β-catenin is characteristic of glioblastoma (GBM) and correlates with poor prognosis. (**A**) Kaplan–Meier plot showing the overall survival of The Cancer Genome Atlas (TCGA) lower grade glioma and glioblastoma cohort (Glioma (GBMLGG), *n* = 1152) according to their histological type. (**B**) β-catenin expression across the different histological type of glioma and glioblastoma in the TCGA GBMLGG cohort. (**C**) Kaplan–Meier plot based on the β-catenin expression level in only the glioblastoma component of the TCGA GBMLGG dataset using the gene expression RNAseq-illuminaHiSeq. Expression cutoff is based on median expression. LGG, low grade glioma; GBM, glioblastoma.

To determine the clinical relevance of the Wnt/β-catenin signaling in GBM, we accessed and analyzed the cancer genome atlas (TCGA) lower grade glioma and glioblastoma cohort (GBMLGG, *n* = 1152) dataset. We observed that of all four histological sub-types of glioma brain tumor in the GBMLGG cohort, namely astrocytoma (*n* = 196), oligoastrocytoma (*n* = 134), oligodendroglioma (*n* = 195), and GBM (*n* = 604), GBM in its various forms exhibit the poorest survival rates (*p* = 0.001, log-rank test = 475.2), with a 100% mortality rate by 4.1, 8.2, and 10 year post-diagnosis for GBM (*n* = 30), treated primary GBM (*n* =20), and untreated primary (de novo) GBM (*n* = 554), respectively (Figure 1A). Furthermore, our analyses revealed the histological subtypes of glioma with the poorest overall survival were characterized by the most aberrantly expressed β-catenin gene expression as demonstrated by a median β-catenin expression of 13.125 in patients with GBM, treated primary GBM and untreated primary (de novo) GBM, compared to 12.80, 12.69, and 12.70 in those with astrocytoma, oligoastrocytoma and oligodendroglioma, respectively (Figure 1B). This is consistent with the high grade, highly malignant, and aggressive character of GBM, compared to the lower grade gliomas. In parallel analyses, we observed that 23.75% and 18.75% of GBM patients with low expression of β-catenin (*n* = 301) were more likely to be alive at the 5- and 10-yr time-point post-diagnosis, respectively, compared to patients with high β-catenin expression (*n* = 303) (*p* = 3.57e-11; log-rank test = 43.8; Figure 1C). These data not only indicate a functional relationship between patients’ survival and the expression levels of β-catenin, but also points to a probable causative or at least participatory role for β-catenin overexpression in the poor prognosis of GBM patients (also see Figure S1).

### 3.2. β-Catenin Facilitates GBM Oncogenicity and Disease Recurrence, as well as Their Cancer Stem Cell-Like Traits

**Figure 2:**
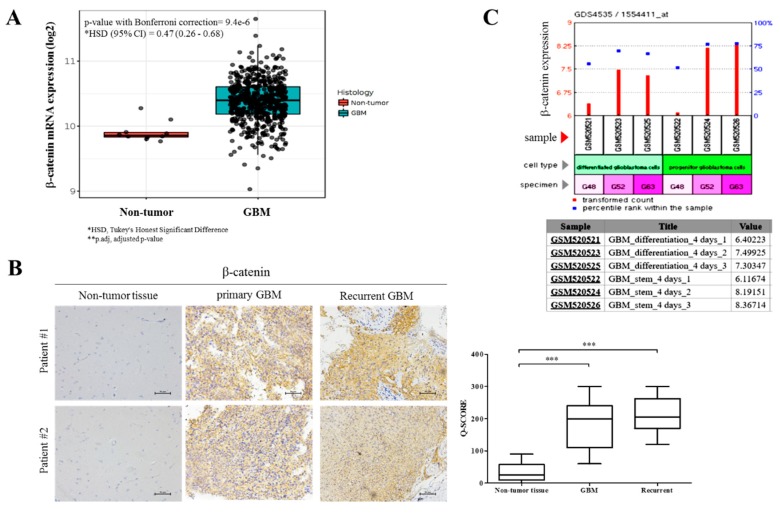
β-catenin facilitates GBM oncogenicity and disease recurrence, as well as their cancer stem cell-like traits. (**A**) TCGA brain dataset (*n* = 557) using the Human Genome U133A Array showing the differential β-catenin mRNA expression in GBM (*n* = 547) and normal brain (*n* = 10) samples. (**B**) Immunohistochemistry (IHC) staining comparing expression of β-catenin in GBM, recurrent and adjacent non-tumor brain tissues in 2 Taipei Medical University Shuang Ho Hospital (TMU-SHH) brain tumor patients. * *p* < 0.05, ** *p* < 0.01, *** *p* < 0.001; GBM, glioblastoma. Scale bar: 50 μm (**C**) GEO dataset GSE20736/GPL570/GDS4535 shows time-dependent enhancement of β-catenin expression levels in human progenitor GBM cells compared to their differentiated counterparts.

To better appreciate the oncogenic role of β-catenin in GBM and investigate its likely causative or participatory role in gliomagenesis and disease course, we accessed and analyzed the TCGA GBM dataset (*n* = 538), for differential β-catenin expression profile. Using the Human Genome U133A Array, compared to the normal brain tissues (*n* = 10), β-catenin (reporter ID 201533_at), was overexpressed in the GBM tissues (*n* = 528; *p* = 9.4e-6), with a Tukey’s honest significant difference (HSD) of 0.47 and 95% confidence interval of 0.26–0.68 (Figure 2A). This data does suggest an initiating or enhancing role for β-catenin in GBM. In confirmatory assays using clinical samples from our GBM cohort, we evaluated and compared the expression of β-catenin in primary GBM, recurrent GBM and non-tumor (“normal”) brain tissue. Our immunohistochemical staining results showed enhanced β-catenin expression in the primary (8-fold, *p* < 0.001) and recurrent GBM (8.1-fold, *p* < 0.001) tissues, compared to the non-tumor tissues (Figure 2B). To further provide insight into the mechanistic underlining of GBM oncogenicity and recurrence, using the SE20736/GPL570/GDS4535 (HG-U133_Plus_2) Affymetrix Human Genome U133 Plus 2.0 Array dataset, we examined the expression profile of β-catenin (1554411_at) in cultures of progenitor (GBM-SCs) and four-day differentiated GBM cells derived from surgical specimens; our data show that compared to the differentiated GBM cells in samples GSM520521, GSM520523, and GSM520525, the cumulative β-catenin expression in the GBM-SCs samples GSM520522, GSM520524, and GSM520526 was significantly higher (Figure 2C). Specifically, as shown in Figure 2C, while the differential β-catenin expression was equivocal between GSM520521 and GSM520522, a 30% and 32.25% enhancement in β-catenin expression was observed in GSM520524 and GSM520526 compared to GSM520523 and GSM520525, respectively. These data indicate the probable involvement of β-catenin expression in facilitating GBM oncogenicity and disease recurrence, as well as their cancer stem cell-like traits.

### 3.3. 4-AAQB Disrupts the CSC-Associated Oncogenic β-Catenin/TCF-1/Stat3 Signaling Axis in GBM Cells

**Figure 3:**
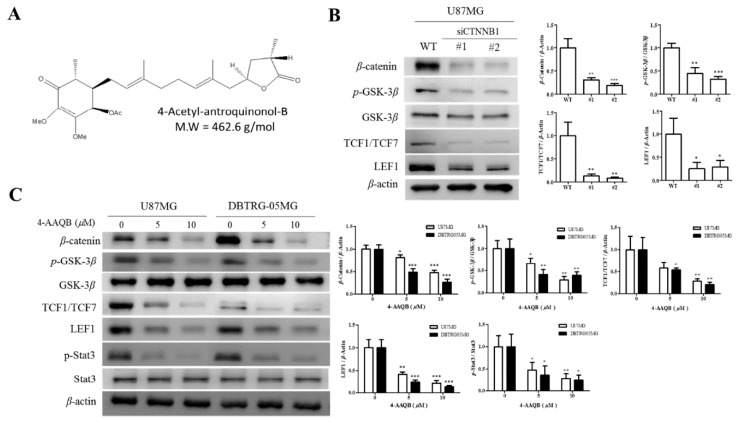
4-AAQB disrupts β-catenin/TCF-1/Stat3 signaling axis in GBM cells. (**A**) Chemical structure of 4-Acetylantroquinonol B; C26H38O7, 462.58 g/mol. Representative Western blot data and graphical quantification showing (**B**) the knock-down efficiency of β-catenin in human GBM cell line U87MG, with corresponding effect on the expression levels of pGSK3β, GSK-3β, TCF1/TCF7, and LEF1 proteins; and (**C**) the dose-dependent downregulation of β-catenin, pGSK3β, TCF1/TCF7, LEF1, and p-Stat3 proteins in U87MG and DBTRG05MG cells treated with increasing concentrations of 4-AAQB for 24 h. β-actin served as loading control. siCTNNB1, short interfering RNA directed specifically against β-catenin; WT, wild type; * *p* <0.05, ** *p* <0.01, *** *p* <0.001. All data is representative of experiments performed 4 times in triplicates.

Having demonstrated the efficacy of 4-AAQB alone or in combination with cisplatin in highly aggressive epithelial cancers, in vitro and in vivo, in earlier work [11], we thus investigated the therapeutic effect and probable β-catenin—mediated efficacy of 4-AAQB (Figure 3A) in the human GBM cell lines—U87MG and DBTRG05MG. Using the Western blot assay, we demonstrated that similar to the inhibitory effect of the transient silencing of β-catenin using short interfering RNA (siRNA) on the protein expression of β-catenin, its upstream modulator p-GSK-3, and downstream effectors TCF1/TCF7 and LEF1 in U87MG cells (Figure 3B), treatment of U87MG and DBTRG05MG cells with 5 and 10 μM 4-AAQB significantly and dose-dependently downregulated the expression of β-catenin, p-GSK-3β, TCF1/TCF7 and LEF1, as well as p-Stat3 (Figure 3C). These data not only corroborate demonstrated convergence of the canonical Wnt/β-catenin and Stat3 signaling pathways [13,14,15], but also is indicative of the therapeutic efficacy of 4-AAQB in GBM cells through disruption of the CSC-associated oncogenic β-catenin/TCF-1/Stat3 signaling axis.

### 3.4. 4-AAQB Inhibits the Nuclear Localization of β-Catenin, Sox2, and Oct4 in GBM Cells

**Figure 4:**
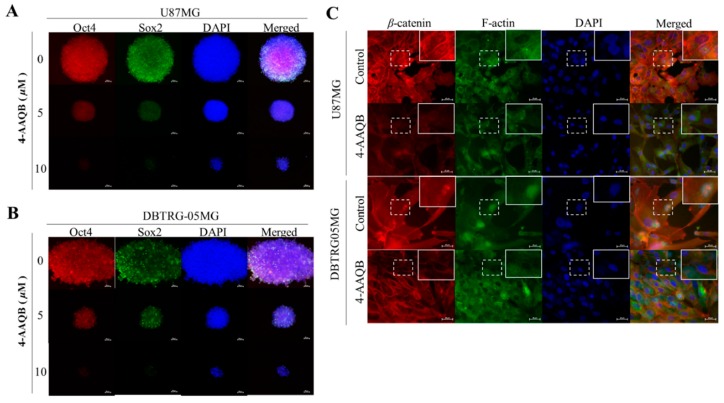
4-AAQB inhibits the nuclear localization of β-catenin, Sox2, and Oct4 in GBM cells. U87MG and DBTRG-05MG cells treated with or without 5–10 μM 4-AAQB were immunostained with Sox2 (green), Oct4 (red), β-catenin (red), and F-actin (green) antibodies, then image visualization and analysis carried out by fluorescence microscopy. Treatment with 5 or 10 μM 4-AAQB markedly decreased nuclear Sox2 and Oct4 protein expression in (**A**) U87MG or (**B**) DBTRG-05MG cells. (**C**) Immunohistochemistry show reduced nuclear expression of β-catenin and F-actin after treatment with 10 μM 4-AAQB. Original magnification ×200. DAPI (blue) served as nuclear marker.

Since there is evidence that the key stemness genes *Nanog*, *Oct4*, and *Sox2* are directly or indirectly regulated in a context-specific and TCF1/TCF3-involved manner by *β*-*catenin* [16], and that the blocking of *β*-catenin nuclear localization with small-molecule inhibitors significantly enhances reprogramming efficiency of stem cells [17], we then evaluated the effect of 4-AAQB on the nuclear localization of β-catenin, Sox2, and Oct4 using the dual-color immunofluorescent staining. Our results demonstrated that 48 h exposure to 5–10 μM 4-AAQB significantly reduced the nuclear expression and co-localization of Sox2 and Oct4 in U87MG (Figure 4A) and DBTRG05MG (Figure 4B) cells, and this correlates with the concurrent decrease in β-catenin and F-actin expression after treatment with 10 μM 4-AAQB (Figure 4C). These data are corroboratory of previous results and demonstrate the therapeutic efficacy of 4-AAQB in GBM cells through disruption of F-actin-mediated essential cellular bioactivities, pluripotency, and CSCs-associated oncogenic β-catenin signaling.

### 3.5. 4-AAQB Significantly Suppresses the Viability and Oncogenicity of GBM Cells

**Figure 5:**
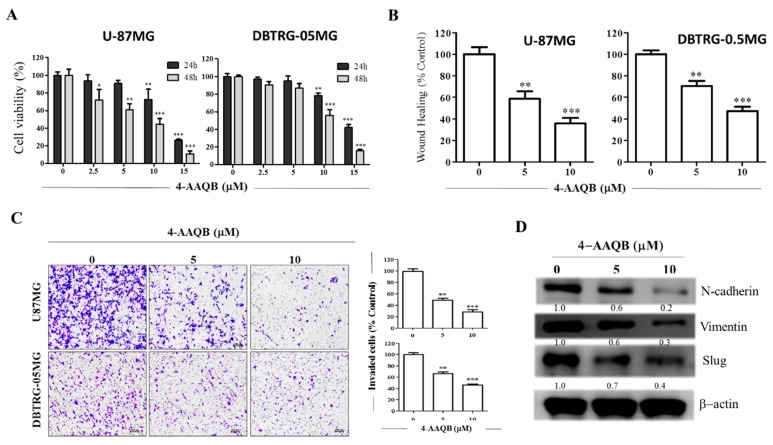
4-AAQB significantly suppresses the viability and oncogenicity of GBM cells. (**A**) The dose-dependent cytotoxic effect of 4-AAQB on the cell viability of U-87MG and DBTRG-05MG. (**B**) Representative photo-images and graphical quantification showing the effect of 5 μM and 10 μM of 4-AAQB on the migration of U-87MG and DBTRG-05MG over 24 h duration. (**C**) 4-AAQB dose-dependently inhibit the number of invaded U-87MG and DBTRG-05MG cells. Scale bar: 50 μm. (**D**) The inhibitory effect of 5 M and 10 M 4-AAQB on the expression levels of β-catenin, vimentin, and slug proteins in DBTRG-05MG cells as shown by Western blot assay. β-actin served as loading control. * *p* < 0.05, ** *p* < 0.01, *** *p* < 0.001; All data is representative of experiments performed 4 times in triplicates.

To further understand the therapeutic relevance of 4-AAQB in GBM, we examined the effect and therapeutic efficacy of 2.5–15 M 4-AAQB in U87MG and DBTRG05MG cells. Our drug cytotoxicity assay results demonstrate that the viability of U87MG and DBTRG05MG cells treated with 4-AAQB was markedly reduced in a dose- and time-dependent manner, compared to the untreated control cells (Figure 5A). Since oncogenic signaling and/or oncogenic transformation of cancer cells is associated with and underlies the acquisition of migratory, invasive, and metastatic phenotypes; we evaluated the effect of 4-AAQB on the migration of GBM cells using the scratch wound healing assay. Confluent U87MG or DBTRG05MG monolayer adherent cells were scratched along the median axis and 4-AAQB- or vehicle-containing culture medium was added, and wound closure monitored over 24 h. The untreated control U87MG or DBTRG05MG cells migrated significantly faster into the scratched area, compared to the cells treated with 5 M or 10 M 4-AAQB (Figure 5B). Using the transwell invasion assay, we demonstrated that when treated with 5 M or 10 M 4-AAQB, the GBM cells were less invasive than their untreated counterparts, with a 52% (*p* < 0.01) or 70% (*p* < 0.001) reduction in number of 5 M or 10 M 4-AAQB-treated invaded U87MG cells, respectively, compared to the untreated control cells. The number of 5 M or 10 M 4-AAQB-treated invaded DBTRG05MG cells was reduced by 35% (*p* < 0.01) or 48% (*p* < 0.001), respectively, in comparison to their untreated counterpart (Figure 5C). To characterize the underlying mechanism for the observed reduction in cell viability, migration and invasion, we evaluated the expression of a selected panel of oncogenic proteins and observed that 5 μM or 10 μM 4-AAQB significantly downregulated the expression levels of β-catenin, vimentin, and slug proteins, in a dose-dependent manner (Figure 5D). These results are indicative of 4-AAQB ability to inhibit GBM cell viability and/or proliferation, migration, invasion, and consequently, deter metastasis in GBM cells.

### 3.6. 4-AAQB Markedly Inhibits the Stem Cell-Like Phenotype of U87MG and DBTRG-05MG Cells by Modulation of β-Catenin Expression

**Figure 6:**
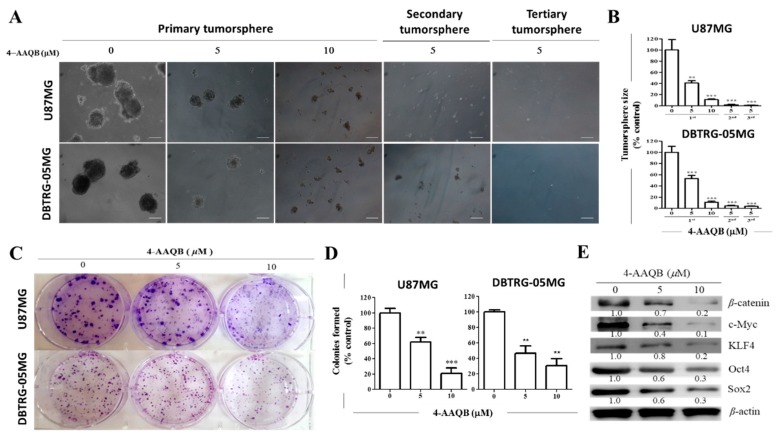
4-AAQB markedly inhibits the stem cell-like phenotype of U87MG and DBTRG-05MG cells. (**A**) Representative photo-images and (**B**) graphical quantification showing 4-AAQB significantly reduce the size and number of U87MG and DBTRG-05MG primary, secondary, and tertiary generation tumorspheres formed. Original magnification = 200×. (**C**) Representative photo-images and (**D**) graphical representations of 4-AAQB effect on the ability of U87MG and DBTRG-05MG cells to form colonies; (**E**) The dose-dependent inhibitory effect of 5–10 M 4-AAQB treatment on the expression levels of β-catenin, c-Myc, KLF4, Oct4, and Sox2 proteins in DBTRG05MG cells shown in a representative Western blot data. β-actin served as loading control. * *p* < 0.05, ** *p* < 0.01, *** *p* < 0.001; All data is representative of experiments performed 4 times in triplicates.

Since undifferentiated GBM-SCs are highly proliferative, invasive, and drug-resistant cells with enhanced colony- and tumorsphere-formation efficiency [18,19,20], compared to the remaining cancer cell population, and are implicated in tumor formation, reduced treatment response, and recurrence [20], we evaluated the effect of 4-AAQB on these cancer stem-cell-like phenotypes in human GBM cell lines, U87MG and DBTRG05MG. Results of our anchorage-independent tumorsphere formation assay demonstrated that treatment with 5 M or 10 M 4-AAQB significantly reduced the tumorsphere-forming capacity of U87MG and DBTRG05MG cells (79–96% reduction, *p* < 0.001) (Figure 6A,B). More interestingly, consistent with our portended self-renewal-limiting potential of 4-AAQB, U87MG- or DBTRG-05MG—derived tumorspheres pre-treated with 5 M 4-AAQB significantly lost their ability to form secondary or tertiary generation of tumorspheres, qualitatively and quantitatively (*p* < 0.001) (Figure 6A,B). Similarly, using the colony formation assay, we demonstrated that the treatment of GBM cells with 4-AAQB inhibits the formation of GBM colonies in a dose-dependent manner, as we observed a 39% (*p* < 0.01) and 80% (*p* < 0.001) reduction in number of colonies formed by U87MG cells treated with 5 or 10 M 4-AAQB, respectively, compared to the untreated cells. For the treated DBTRG05MG cells, a 58% (*p* < 0.01) and 64% (*p* < 0.001) inhibition in capacity to form colonies was noted after treatment with 5 or 10 μM 4-AAQB, respectively, compared to their untreated counterparts (Figure 6C,D). These findings were corroborated by Western blot data showing marked downregulation in the expression levels of β-catenin, and the stem cell markers, Oct4, Sox2, c-Myc and KLF4 proteins in a dose-dependent manner when treated with 5 and 10 M 4-AAQB (Figure 6E). These results do demonstrate that 4-AAQB effectively inhibits the stem cell-like phenotype and attenuates the self-renewal capacity of GBM cells and is consistent with the significant downregulation in expression or marked decrease in nuclear localization of β-catenin, and the stemness marker, Sox2 and Oct4 demonstrated earlier in Figure 3 and Figure 4.

### 3.7. Compared to Oral Gavage, Intraperitoneal 4-AAQB Significantly and More Efficiently Suppresses GBM Stem Cell-Induced Tumor Growth, in Vivo

**Figure 7:**
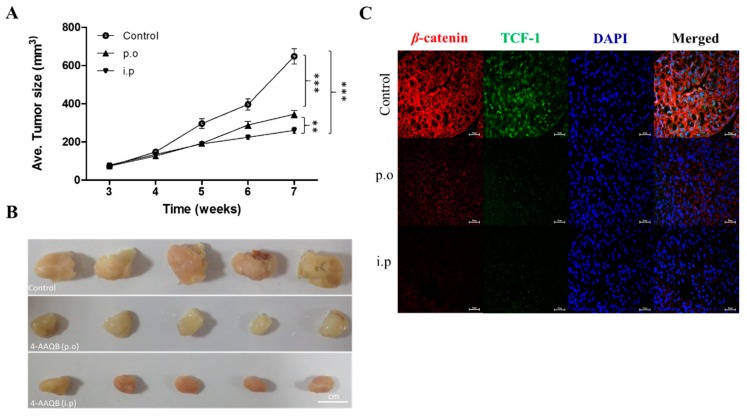
Compared to oral gavage, intraperitoneal 4-AAQB significantly and more efficiently suppresses GBM stem cell-induced tumor growth, in vivo. (**A**) Tumor size vs time curve show the inhibitory effect of 4-AAQB on U87MG tumor growth either via p.o or i.p route, as compared to the control group. (**B**) Photographs of tumor samples harvested from the in vivo studies. (**C**) The differential effects of oral and intraperitoneal administration of 4-AAQB on the expression and localization of TCF-1 and β-catenin in xenograft-derived GBM primary culture. * *p* < 0.05, ** *p* < 0.01, *** *p* < 0.001; 4′, 6′-diamidino-2-phenylindole (DAPI) served as nuclear marker.

Considering the enhanced tumor-initiating capacity of undifferentiated GBM-SCs and their implication in poor treatment response and tumor recurrence [18,19,20], we evaluated the effect of 4-AAQB administered through different routes, namely intraperitoneally (i.p) or orally (p.o) on these CSC-like phenotypes of GBM cells using U87MG single cell solution dissociated from formed tumorspheres, with the aim of validating our in vitro results in vivo. Subcutaneous injection of U87MG cells resulted in tumor formation in all 15 nude mice, which were thereafter treated with PBS, 4-AAQB (i.p), or 4-AAQB (p.o) from day 7 after tumor cell inoculation. No animals died during the treatment. We observed no dyscrasia nor significant difference in body weight between the 3 treatment groups before and after the experiments, neither was any metastases found in organs of the thoracic or abdominal cavity. Tumor growth curves showed that the average size of tumors in the 4-AAQB (i.p) group was significantly smaller compared to the 4-AAQB (p.o) (163.6 ± 78.4 mm^3^ vs. 196.4 ± 108.9 mm^3^, *p* = 0.0095) or PBS-treated control groups (163.6 ± 78.4 mm^3^ vs. 307.0 ± 231.4 mm^3^, *p* = 0.0001) (Figure 7A,B). Similarly, our immunoflourescence staining showed that compared with data from mice subjected to oral gavage of 4-AAQB (p.o), intraperitoneal injection of 4-AAQB (i.p) significantly decreased the nuclear and cytomenbranous expression of both TCF-1 and β-catenin, as well as inhibited their nuclear co-localization in the xenograft-derived GBM primary culture (Figure 7C). These findings indicate that 4-AAQB treatment, especially the intraperitoneal administration, inhibits tumor growth effectively.

## 4. Discussion

β-catenin is an essential transcription co-activator of the Wnt/β-catenin signaling pathway. Inappropriate activation and/or expression of the β-catenin induces aberrant activation of the canonical Wnt pathway in various cancer types including brain tumors and are in fact implicated in the enhanced proliferation and evasion of cell death of GBM cells [21], and poor prognosis in GBM patients [22]. While it has been suggested that β-catenin expression is a prognostic marker in GBM, for the first time, in a comparative analysis of different histological types of glioma, we showed that the aberrant expression of β-catenin is most characteristic of GBM cells and correlates with shorter overall and relapse-free survival (Figure 1). These findings are interesting in the context of our data showing overexpression of β-catenin in primary and recurrent GBM relative to normal brain tissues (Figure 2), which is corroboratory to the findings of Liu X, et al. [21] in which higher expression level of β-catenin was found in astrocytic glioma patients with high grade in comparison with the normal controls, while siRNA-mediated silencing of β-catenin inhibited the proliferation and resulted in apoptosis of human U251 cells, with arrested cell cycle in G_0_/G_1_. Against the background of β-catenin overexpression in recurrent GBM cells, of particular clinical relevance is our data showing cumulative enhanced expression of β-catenin in undifferentiated (progenitor) GBM-SCs compared to the differentiated GBM (Figure 2), as it not only suggests a critical role for β-catenin in the resistance of GBM cells to therapy and disease recurrence, which are characteristic of CSCs, thus projecting β-catenin as a putative biomarker for GBM initiating cells or GBM-SCs. GBM-SCs exhibit distinct phenotypic, histopathologic and molecular features; they are highly tumorigenic, differentiate into heterogeneous glioma cell types or self-renew to sustain the GBM-SC pool, thus, facilitating and enhancing gliomagenesis [5]. These features make the eradication of these β-catenin-rich GBM-SCs a crucial therapeutic strategy for the effective treatment of GBM [23].

4-Acetylantroquinonol B (4-AAQB), an acetylated form of antroquinonol, a bioactive mycelial isolate of *Antrodia cinnamomea*, which is a Taiwanese mushroom with documented anti-inflammatory, blood sugar-lowering, vascular tone-relaxing, anti-proliferative, anti-metastasis, and autophagy-modulating activity [10,11,24]. Following the demonstrated CSCs-limiting anticancer efficacy of 4-AAQB in different cancer types, including colorectal, hepatocellular, breast and ovarian carcinomas, by our team and others [10,11,24], we herein investigated and demonstrated for the first time to the best of our knowledge, the therapeutic effect of 4-AAQB against GBM cells, by targeting GSM-SCs through the dysregulation of the canonical Wnt signaling pathway in a β-catenin-mediated manner. The canonical Wnt signaling pathway, especially mediated by aberrant or ectopic expression of a constitutively-active β-catenin, is broadly known to be involved in epithelial-to-mesenchymal transition (EMT), increased cell motility and tumor invasion, which are elements of tumor metastasis and contribute largely to cancer-related deaths [6], thus, our findings showing that treatment with 4-AAQB significantly inhibit the viability, migration, and invasive potential of GBM cells, while concurrently suppressing the expression levels of β-catenin, p-Stat3, vimentin, and slug proteins (Figure 3 and Figure 5) has clinical implications and is consistent with research reports that the inhibition of β-catenin signaling disrupts the Wnt pathway, inhibits the downstream TCF/LEF transcriptional activity and suppress oncogenic activity [25,26]; more so we demonstrated that this suppression of GBM cell oncogenicity is associated with the concurrent marked reduction in the expression of nuclear β-catenin, Sox2, and Oct4, as well as inhibited nuclear co-localization of β-catenin and F-actin in the GBM cells (Figure 3 and Figure 4), which is consistent with contemporary knowledge that the Wnt/β-catenin signaling plays with vital role in embryogenesis, activates embryonic stem cells (ESCs), regulates adult stem cells (ASCs), and modulates CSC biology by modulating the expression and activity of pluripotency and self-renewal transcription factor, namely Nanog, Oct4, Sox2, c-Myc [27].

Interestingly, in corroborating assays, we showed that 4-AAQB significantly diminished the colony-forming and tumorsphere-forming population, as well as the self-renewal capacity of GBM cells in a dose-dependent manner (Figure 6). Tumorspheres are characterized by enhanced anchorage-independent clonogenicity and clonal expansivity [28], thus their use as in vitro representation of the human CSC model. The GBM-SCs are implicated in tumorigenesis, therapy resistance, metastasis, and GBM recurrence, making them crucial molecular targets for effective anti-GBM therapeutic strategy, thus, the ability of 4-AAQB to effectively target this cellular subset is therapeutically-relevant in the light of the short-lived response of GBM patients to standard anti-GBM therapy consisting of temozolomide (TMZ) and radiation, which is almost always followed by recurrence, and not unconnected with the presence of unscathed treatment-insensitive GBM-SCs [5,18,19,20,23,27]. Results of our tumor xenograft in vivo studies are corroboratory of our in vitro findings, as they showed that treatment with 4-AAQB significantly suppress GBM-SC-induced tumor growth, in vivo. More so, we observed that compared to oral gavage, the intraperitoneal administration of 4-AAQB not only significantly, but also more efficiently suppressed the formation and growth of tumors in the GBM mice models (Figure 7). This is clinically relevant as it informs clinical decision making on the optimal route of drug administration in the treatment of GBM. As with the experimental design of in vivo animal studies, the administration of therapeutic compound in human is a critical component of every therapeutic strategy, as the optimization of drug delivery while minimizing likely adverse effects may constitute a deciding factor in treatment failure or response, and the degree or extent of such response [29]. Consistent with our observation, parenteral administration of therapeutics, specifically intraperitoneally, typically exhibits enhanced bioavailability of the drug because this route avoids the first-pass effect of liver metabolism, as commonly witnessed with oral administration of therapeutics; Similarly, the i.p route circumvents the unpredictability often associated with enteral absorptive processes [30]. Our results highlight the therapeutic potential of 4-AAQB for anti-GBM treatment, especially for reversal of GBM stem cell-associated TMZ resistance and lay the groundwork for further pre-clinical exploration and clinical utility of 4-AAQB for enhancement of TMZ efficacy in hitherto therapy-resistant GBM cells by either sensitizing GBM-SCs to TMZ or potentiating the anticancer effect of TMZ.

## 5. Conclusions

In conclusion, for the first time to the best of our knowledge, as shown in our Graphical Abstract, we demonstrate that 4-AAQB effectively targets the often unscathed chemoradiotherapy, insensitive GBM-SCs, and inhibits the not-too-innocent differentiated bystander GBM cells by suppressing β-catenin, inhibiting Stat3 activation and disrupting the canonical Wnt signaling pathway, thus projecting 4-AAQB as a novel small-molecule inhibitor of the Wnt/β-catenin signaling pathway with GBM-SC -targeting potentials, Thus projecting 4-AAQB as a putative therapeutic agent for effective anti-GBM target therapy with the promise of improving survival.

## Supplementary Materials

The following are available online at http://www.mdpi.com/2072-6694/10/12/491/s1, Figure S1: Aberrant expression of β-catenin is characteristic of GBM and correlates with poor prognosis.
