# Supplementary material for: The Disruption of the β-Catenin/TCF-1/STAT3 Signaling Axis by 4-Acetylantroquinonol B Inhibits the Tumorigenesis and Cancer Stem-Cell-Like Properties of Glioblastoma Cells, In Vitro and In Vivo
Source: Cancers (Basel). 2018 Dec 5;10(12):491. doi: 10.3390/cancers10120491 (PMC6315804; doi:10.3390/cancers10120491)
Supplement: Supplementary file 1 [file cancers-10-00491-s001.pdf]

Supplementary Materials: The Disruption of the β-catenin/TCF-1/Stat3 Signaling Axis by 4-Acetylantroquinonol B Inhibits the Tumorigenesis and Cancer Stem Cell-Like Properties of Glioblastoma Cells, In Vitro and In Vivo

Heng-Wei Liu, Yu-Kai Su, Oluwaseun Adebayo Bamodu, Dueng-Yuan Hueng, Wei-Hwa Lee, Chun-Chih Huang, Li Deng, Michael Hsiao, Ming-Hsien Chien, Chi-Tai Yeh, Chien-Min Lin

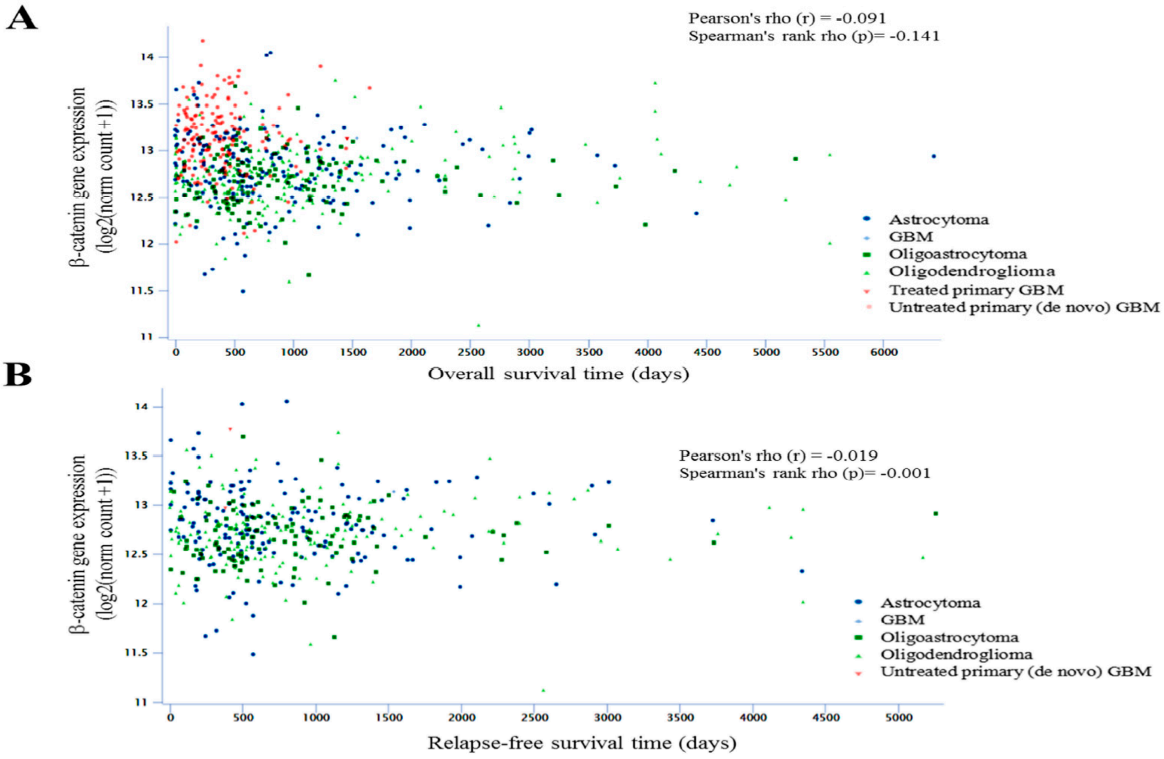

Figure S1. Aberrant expression of β-catenin is characteristic of GBM and correlates with poor prognosis. Analyses of the TCGA lower grade glioma and glioblastoma cohort (GBMLGG, n = 1152) according to their histological type show that (**A**) β-catenin is notably more expressed and correlates more with worse overall survival in untreated primary GBM, GBM, and treated primary GBM, compared to astrocytoma, oligoastrocytoma and oligodendroglioma. (**B**) β-catenin is notably more expressed and correlates more with worse relapse-free survival in untreated primary GBM, and GBM, compared to astrocytoma, oligoastrocytoma and oligodendroglioma.

© 2018 by the authors. Licensee MDPI, Basel, Switzerland. This article is an open access article distributed under the terms and conditions of the Creative Commons Attribution (CC BY) license (http://creativecommons.org/licenses/by/4.0/).

Cancers **2018**, *10*, 491, doi:10.3390/cancers10120491
